# Shifting Demographics and Polysubstance Drivers of Overdose Mortality in Pennsylvania (1999-2020)

**DOI:** 10.7759/cureus.109871

**Published:** 2026-05-29

**Authors:** Ayushma Acharya, Anthony Donato

**Affiliations:** 1 Internal Medicine, Helping Hands Community Hospital, Kathmandu, NPL; 2 Internal Medicine, Reading Hospital-Tower Health, Reading, USA

**Keywords:** benzodiazepines, opioids, overdose, polysubstance use, race/ethnicity, stimulants

## Abstract

Background

Overdose mortality in the United States has shifted from prescription opioids toward polysubstance use, with mounting evidence of widening racial and ethnic disparities over time. Pennsylvania is among the most heavily impacted states, yet state-level trends by age, race/ethnicity, and substance involvement remain incompletely characterized.

Methods

We analyzed mortality data from the CDC’s Multiple Cause of Death (MCOD) database (1999-2020) among Pennsylvania residents aged ≥15 years. Crude death rate per 100,000 was calculated for overdose by 10-year age groups, and age-adjusted rates were estimated by race/ethnicity. Deaths involving individual substances, including opioids, cocaine, and benzodiazepines, were examined. Temporal trends were assessed using joinpoint regression to identify periods of significant change in the annual percent change in mortality.

Results

Overdose mortality increased substantially across all adult age groups, with the steepest rises among adults aged 25-54 years. Age-adjusted overdose mortality rose in all racial and ethnic groups, but non-Hispanic Black residents experienced the largest relative growth, with rates exceeding those of non-Hispanic White residents by 2020. Hispanic populations also saw substantial increases from low baseline levels. Deaths involving opioids, cocaine, and benzodiazepines all rose sharply, consistent with an increasingly lethal polysubstance environment.

Conclusion

Rapidly escalating overdose deaths in Pennsylvania, concentrated among young and middle-aged adults and disproportionately affecting non-Hispanic Black residents, point to a crisis driven by a volatile polysubstance drug supply. These patterns highlight the need for real-time surveillance and equity-focused, community-led interventions that address both structural inequities and the specific risks posed by opioids, stimulants, and sedative co-use.

## Introduction

In the past two decades, opioid-related mortality has risen sharply in the United States, creating a public health burden. Between 2001 and 2016, deaths increased by 345%, accounting for 1.5% of total deaths in 2016 and over 1.6 million years of life lost (YLL) that year [1,2]. The crisis began with prescription misuse but has shifted to heroin and synthetic opioids such as illicit fentanyl [3,4]. Recently, polysubstance overdose deaths, including fentanyl, cocaine, and benzodiazepines, have also increased [5,6].

These shifts have disproportionately affected certain populations. National data reveal widening racial and ethnic disparities in overdose mortality, particularly among adolescents and young adults [7]. While these trends are well-documented at the national level, less is known about how the epidemic unfolds within individual states. Pennsylvania is among the most severely affected states. However, detailed data disaggregated by age, race/ethnicity, and substance combinations over time are scarce. Further complicating state-level analysis, recent evidence indicates that overdose survival rates may vary by geography and demographic factors due to differences in naloxone administration, making it harder to interpret mortality patterns in geographic regions [8]. The lack of granular data may therefore impede public health efforts by obscuring key drivers of mortality and emerging at-risk groups.

Understanding these local dynamics is particularly important in the current era of fentanyl. As the rapid contamination of drug supplies and the increased co-ingestion of stimulants have diminished traditional surveillance and intervention strategies, the need for novel approaches is evident. Therefore, continued reliance on frameworks focused on prescription opioids fails to address the complexity of the present overdose landscape.

To address these gaps, this study examines temporal trends in overdose mortality in Pennsylvania from 1999 to 2020. The focus is on demographic patterns (age and race/ethnicity) and substance involvement (opioids, cocaine, and benzodiazepines). The analysis utilizes the CDC Wide-ranging Online Data for Epidemiologic Research (WONDER) mortality database to assess the evolution of the epidemic across person, place, and time.

## Materials and methods

Data source

De-identified mortality data were obtained from the CDC Wide-ranging Online Data for Epidemiologic Research (CDC WONDER) Multiple Cause of Death (MCOD) database, covering the period from 1999 to 2020. As all data were de-identified and publicly accessible, this study was exempt from institutional review board oversight [9].

Study population

The analysis included Pennsylvania residents aged 15 and older. Drug overdose deaths were identified using International Classification of Diseases, Tenth Revision (ICD-10) codes X40-X45 (unintentional), X60-X65 (suicide), X85 (homicide), and Y10-Y15 (undetermined intent). Substances of interest were opioids (T40.0-T40.4 and T40.6), cocaine (T40.5), and benzodiazepines (T42.4) as underlying or contributing causes. Polysubstance combinations included opioids with stimulants (T40.5 and T43.6), benzodiazepines (T42.4), and alcohol (T51.0-T51.9).

Outcome measures

Crude mortality rates per 100,000 population were calculated and stratified by 10-year age cohorts. Age-adjusted mortality rates for racial/ethnic subgroups were estimated using the direct standardization method based on the 2000 US standard population. Population estimates used as denominators for crude and age-adjusted mortality rate calculations were obtained directly through the CDC WONDER MCOD database, which incorporates US Census Bureau population estimates for mortality rate calculations. Racial/ethnic groups included non-Hispanic White, non-Hispanic Black, and Hispanic individuals. Due to limited numbers of deaths among other racial/ethnic groups (such as non-Hispanic Asian, Pacific Islander, and American Indian/Alaska Native), these categories were excluded from trend analyses and not combined into a broader “non-Hispanic other” group. This approach maintained focus on populations with sufficient data to support stable mortality rate estimates and joinpoint trend analysis and avoided distortion or suppression due to small counts.

Statistical analysis

Temporal trends in overdose mortality were analyzed using joinpoint regression analysis (Joinpoint Regression Program, version 4.9.1.0, National Cancer Institute, Bethesda, MD). This method identifies inflection points, or “joinpoints,” where statistically significant changes in the annual percentage change (APC) in mortality occur. Models were fit using log-transformed mortality rates with calendar year as the independent variable. A minimum of 0 and a maximum of 4 joinpoints were permitted based on the number of annual observations available. Model selection was performed using the weighted Bayesian information criterion (weighted BIC) approach. APCs and 95% confidence intervals were reported for each trend segment. Analyses were conducted separately by substance type, age cohort, and racial/ethnic group.

## Results

Temporal trends in overdose mortality by age group

**Figure 1:**
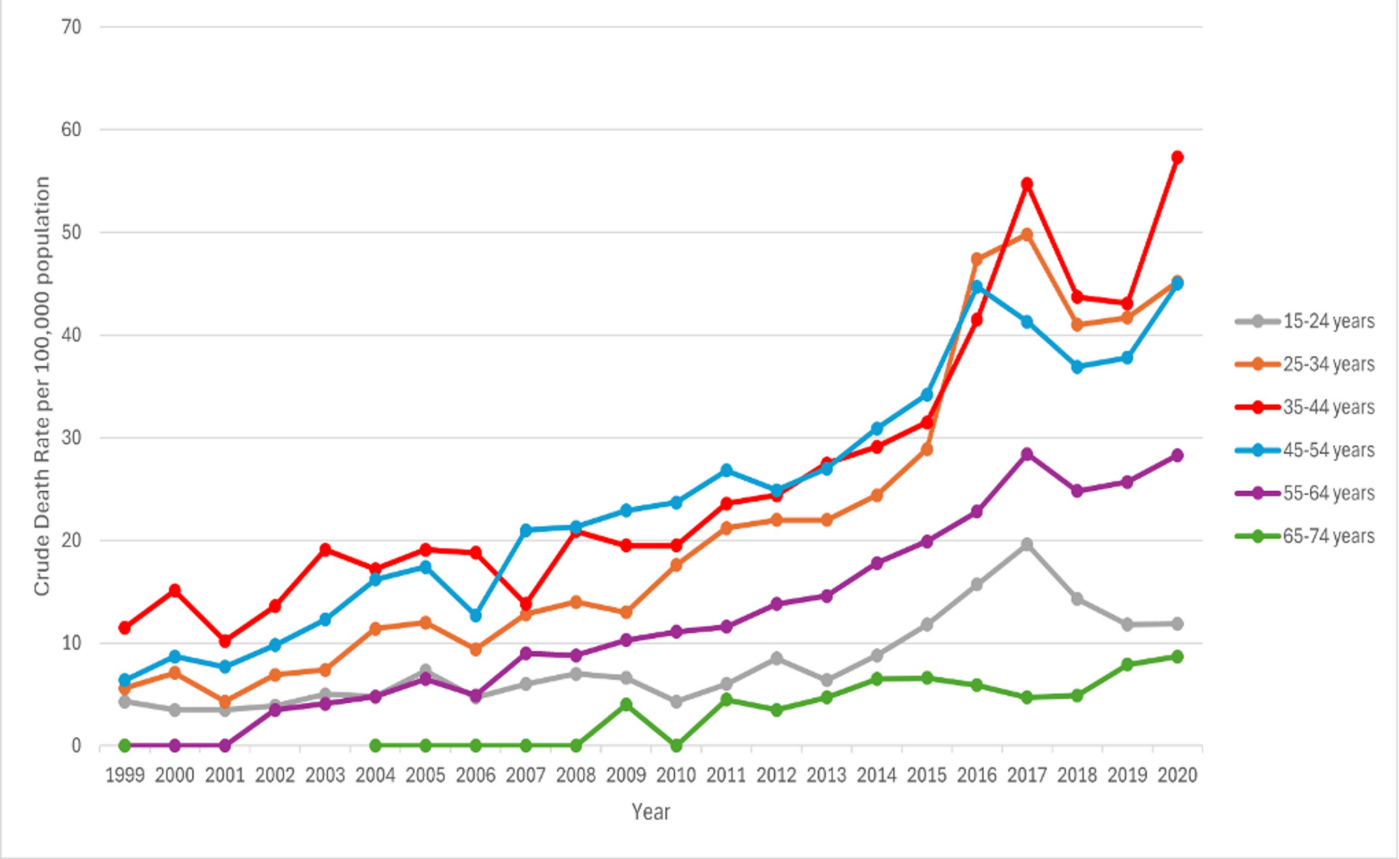
Trend of drug overdose death by age group in Pennsylvania from 1999 to 2020.

**Figure 2:**
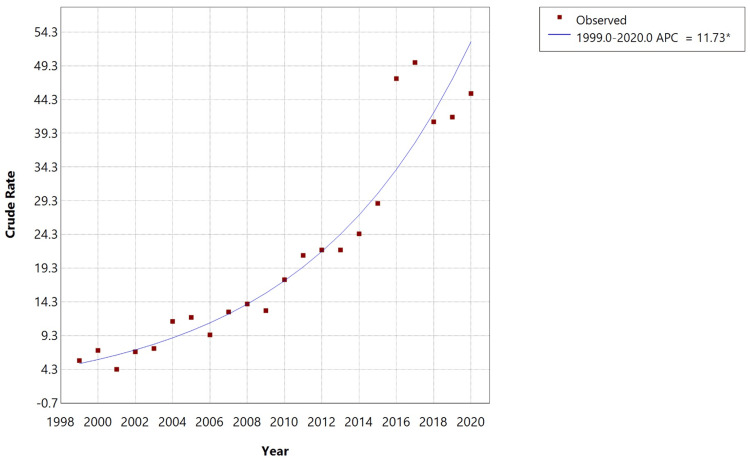
Annual percent change in overdose mortality among adults aged 25-34 years in Pennsylvania (1999-2020). Final selected model: 0 joinpoints. *The annual percent change (APC) is significantly different from zero at the alpha = 0.05 level.

**Figure 3:**
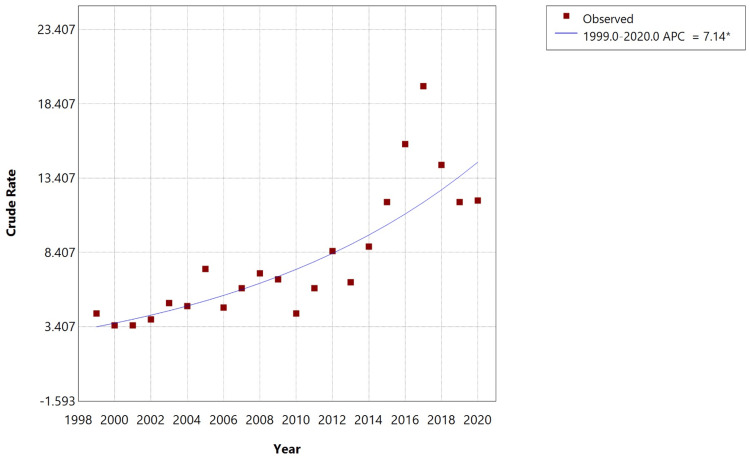
Annual percent change in overdose mortality among adults aged 15-24 years in Pennsylvania (1999-2020). Final selected model: 0 joinpoints. *The annual percent change (APC) is significantly different from zero at the alpha = 0.05 level.

From 1999 to 2020, drug overdose mortality increased substantially in Pennsylvania across all adult age groups (Figure 1). The most pronounced rise occurred among adults aged 25-34 years, with rates increasing from 5.6 to 45.2 per 100,000, corresponding to an annual percent change (APC) of +11.7% (p < 0.05) (Figure 2). Among individuals aged 15-24 years, overdose mortality increased significantly (APC, +7.1%; p < 0.05) (Figure 3).

**Figure 4:**
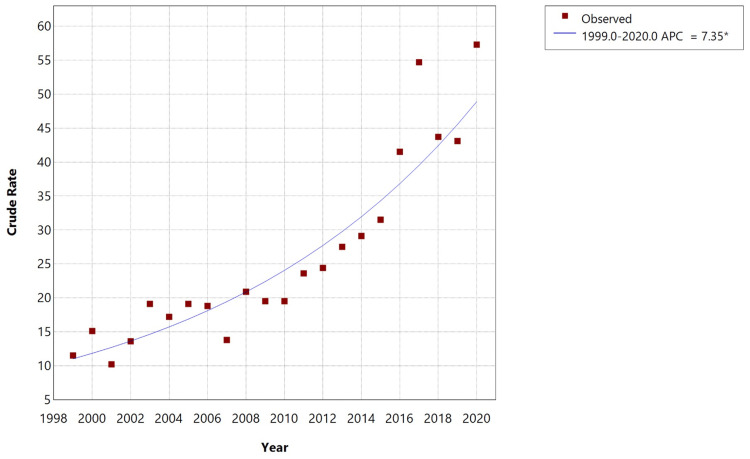
Annual percent change in overdose mortality among adults aged 35-44 years in Pennsylvania (1999-2020). Final selected model: 0 joinpoints. *The annual percent change (APC) is significantly different from zero at the alpha = 0.05 level.

**Figure 5:**
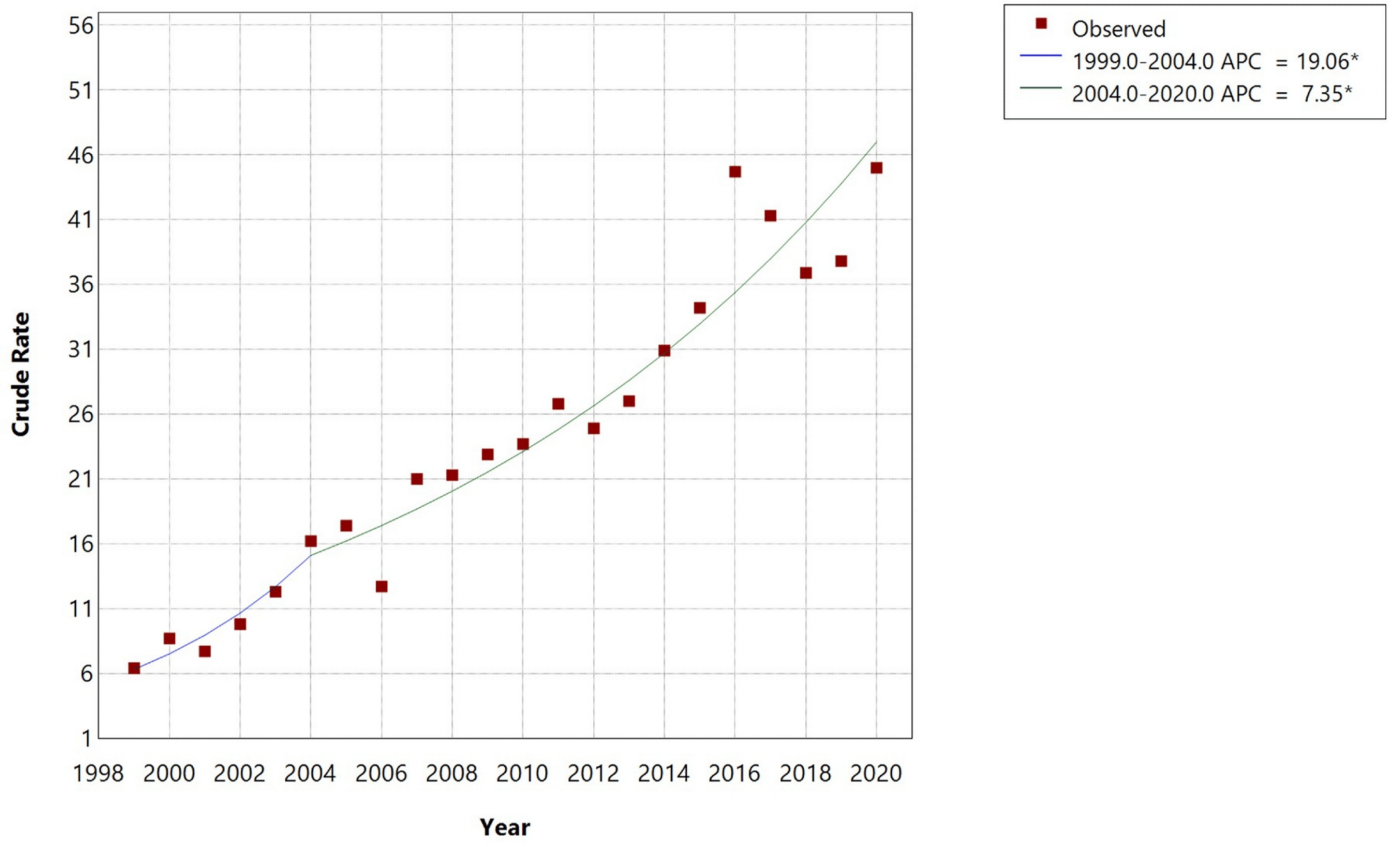
Annual percent change in overdose mortality among adults aged 45-54 years in Pennsylvania (1999-2020). Final selected model: 1 joinpoint. *The annual percent change (APC) is significantly different from zero at the alpha = 0.05 level.

Adults aged 35-44 years experienced a significant increase in mortality (APC, +7.4%; p < 0.05), with rates exceeding 45 per 100,000 by 2020. In the 45-54-year cohort, mortality rose sharply from 1999 to 2004 (APC, +19%; p < 0.05), followed by continued growth from 2004 to 2020 (APC, +7.4%; p < 0.05) (Figures 4, 5).

Racial and ethnic trends

Between 1999 and 2020, age-adjusted overdose mortality rates increased substantially across all racial and ethnic groups in Pennsylvania.

Among non-Hispanic Black individuals, overdose mortality increased from 12.9 deaths per 100,000 population in 1999 to 66.3 per 100,000 in 2020, representing more than a fivefold increase. Rates rose gradually through the early 2000s and then accelerated sharply around 2014, with the steepest increases occurring after 2015.

In comparison, non-Hispanic White individuals experienced an increase in overdose mortality from 7.7 per 100,000 in 1999 to 41.8 per 100,000 in 2020. Although mortality rates among non-Hispanic White individuals remained lower than those of non-Hispanic Black individuals by the end of the study period, both groups demonstrated parallel upward trends during the late 2010s.

**Figure 6:**
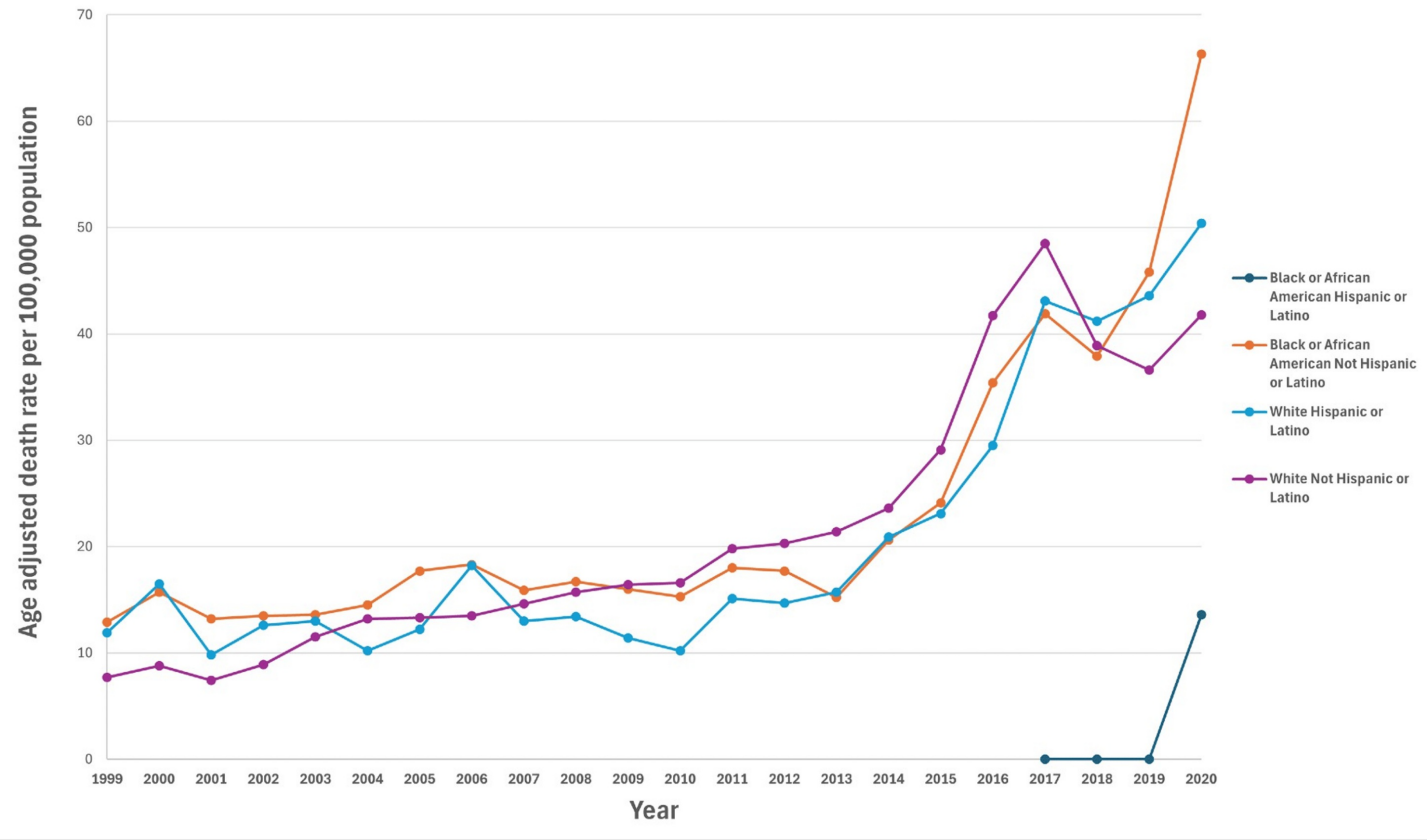
Trend in death from drug overdose per race and ethnicity in Pennsylvania from 1999 to 2020.

Among White Hispanic individuals, mortality rose from 11.9 per 100,000 in 1999 to 50.4 per 100,000 in 2020, with particularly sharp increases after 2015. Rates among Black Hispanic individuals remained the lowest overall but increased from 0-1 per 100,000 in earlier years to 13.6 per 100,000 by 2020 (Figure 6).

Opioid-related mortality

**Figure 7:**
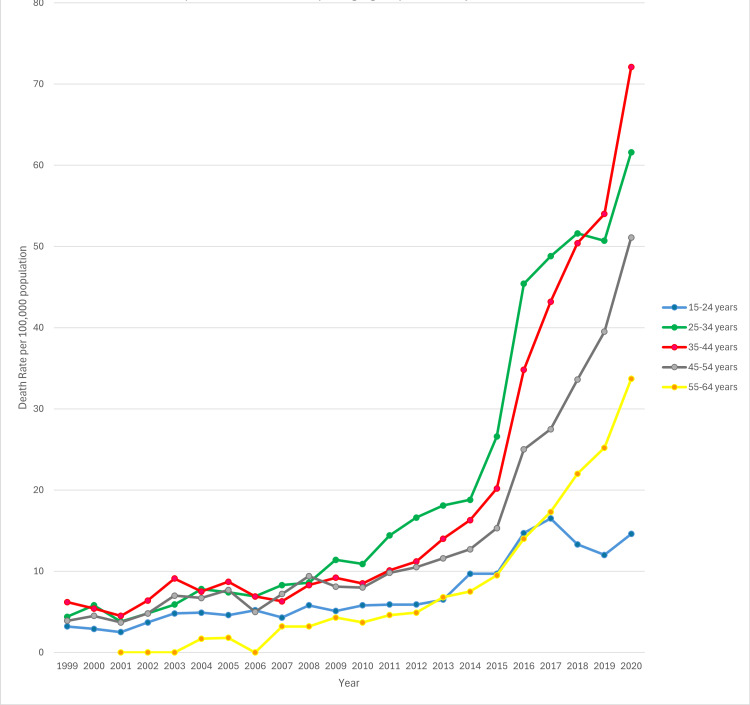
Trends in opioid-related deaths per age group in Pennsylvania from 1999 to 2020.

**Figure 8:**
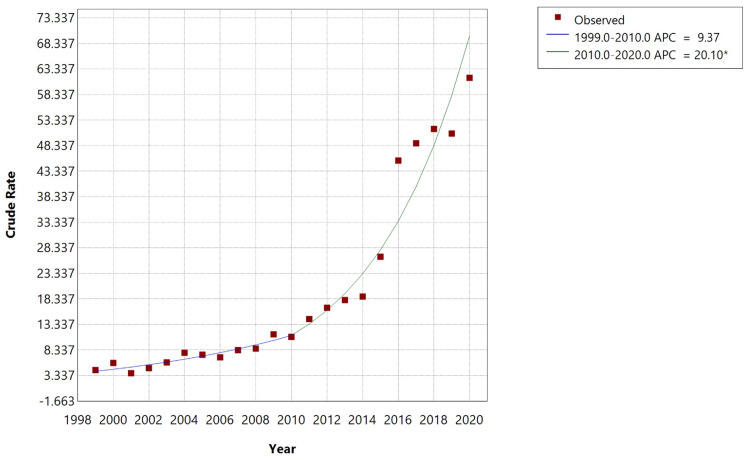
Annual percent change in opioid overdose mortality among adults aged 25-34 years in Pennsylvania (1999-2020). Final selected model: 1 joinpoint. *The annual percent change (APC) is significantly different from zero at the alpha = 0.05 level.

**Figure 9:**
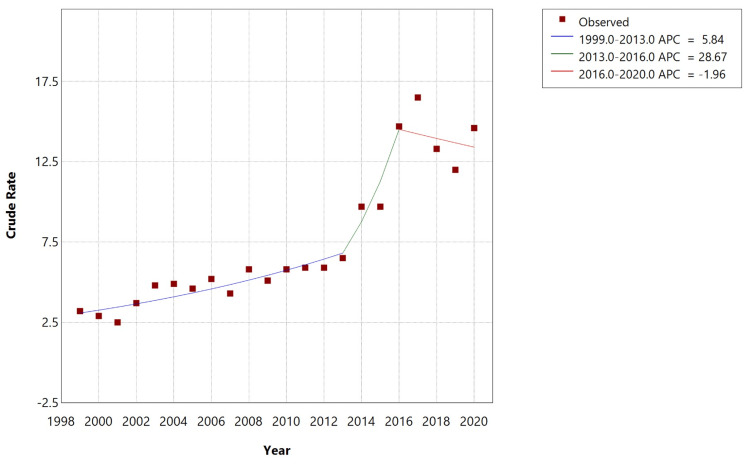
Annual percent change in opioid overdose mortality among adults aged 15-24 years in Pennsylvania (1999-2020). Final selected model: 2 joinpoints.

**Figure 10:**
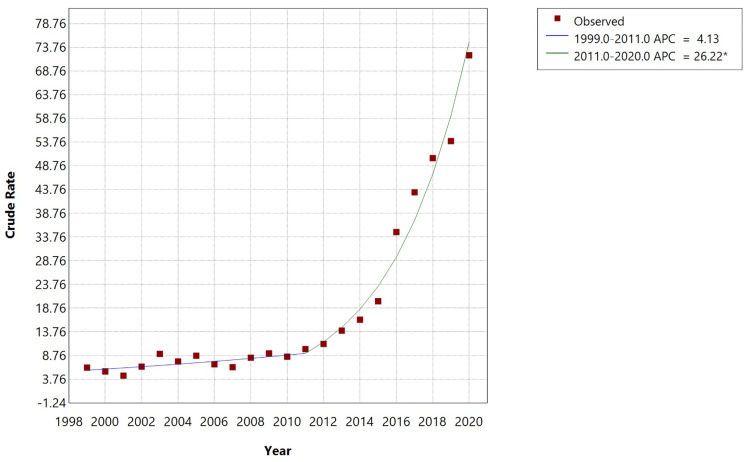
Annual percent change in opioid overdose mortality among adults aged 35-44 years in Pennsylvania (1999-2020). Final selected model: 1 joinpoint. *The annual percent change (APC) is significantly different from zero at the alpha = 0.05 level.

**Figure 11:**
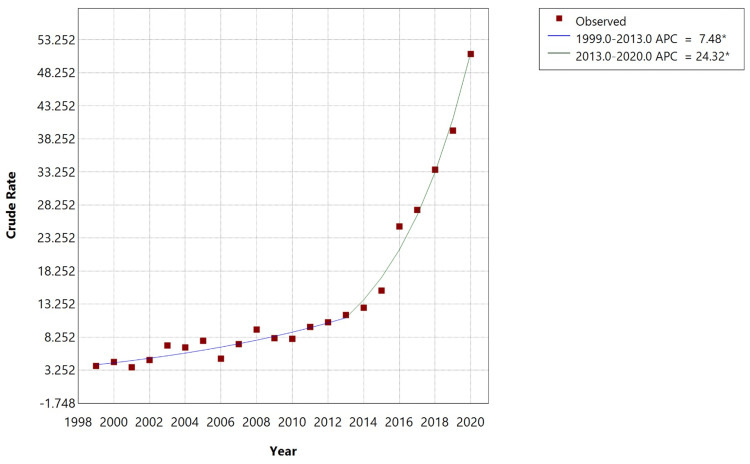
Annual percent change in opioid overdose mortality among adults aged 45-54 years in Pennsylvania (1999-2020). Final selected model: 1 joinpoint. *The annual percent change (APC) is significantly different from zero at the alpha = 0.05 level.

Opioid-related mortality among adults aged 25-34 years increased 14-fold over the study period. Segmented analysis indicated a non-significant steady rise from 1999 to 2010 (APC, +9.4%; p > 0.05), followed by an acceleration between 2010 and 2020 (APC, +20.1%; p < 0.05) (Figures 7, 8). In the 15-24-year group, opioid-related mortality experienced a modest rise from 1999 to 2013 (APC, +5.8%; p > 0.05), a sharp increase from 2013 to 2016 (APC, +28.7%; p > 0.05), and a non-significant plateau thereafter (APC, -1.9%; p > 0.05) (Figure 9). Among individuals aged 35-44 years, opioid mortality rose markedly from 2011 to 2020 (APC, +26.2%; p < 0.05), with rates exceeding 70 per 100,000 by 2020. Similarly, the 45-54-year group experienced a two-phase increase: a steady rise from 1999 to 2013 (APC, +7.5%; p < 0.05), followed by an accelerated trend from 2013 to 2020 (APC, +24.3%; p < 0.05) (Figures 10, 11).

Trends in cocaine- and benzodiazepine-related mortality

**Figure 12:**
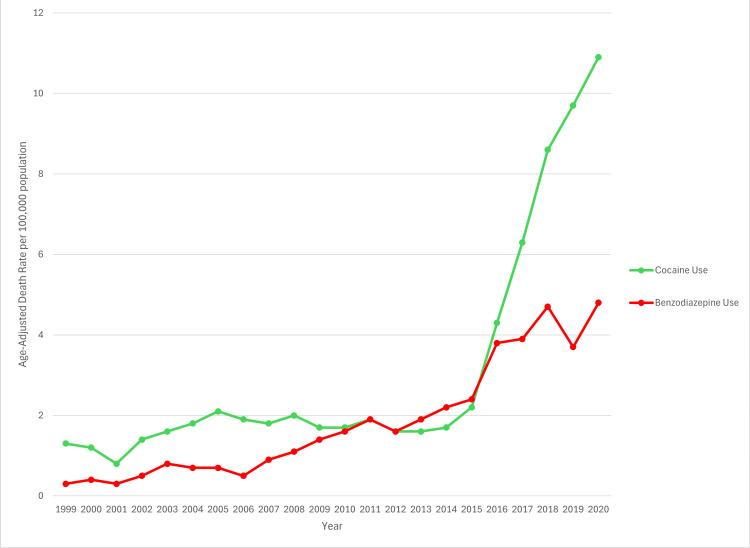
Trends in cocaine and benzodiazepine overdose death in Pennsylvania from 1999 to 2020.

**Figure 13:**
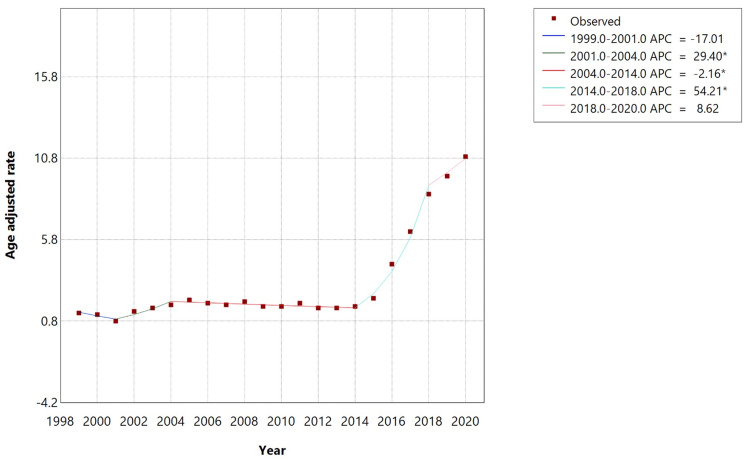
Annual percent change in cocaine overdose mortality among adults in Pennsylvania (1999-2020). Final selected model: 4 joinpoints. *The annual percent change (APC) is significantly different from zero at the alpha = 0.05 level.

**Figure 14:**
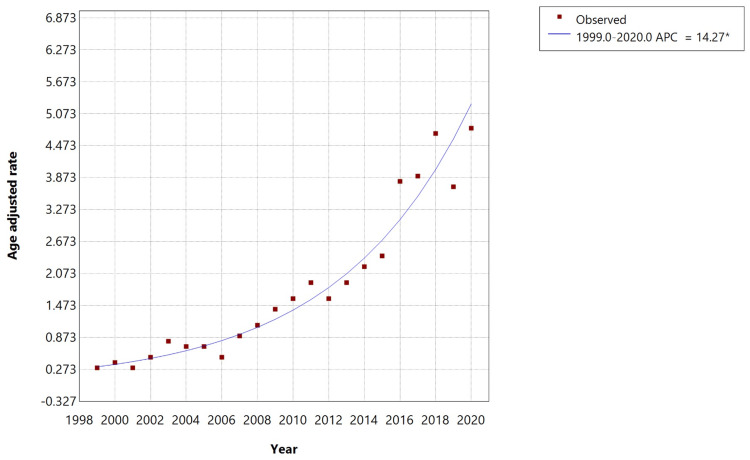
Annual percent change in benzodiazepine overdose mortality among adults in Pennsylvania (1999-2020). Final selected model: 0 joinpoints. *The annual percent change (APC) is significantly different from zero at the alpha = 0.05 level.

Cocaine-related deaths remained stable throughout 2014 but increased sharply thereafter, reaching 10.9 per 100,000 by 2020. From 1999 to 2001, mortality rates declined modestly, although statistically non-significant (APC, -17.0%; p > 0.05). An increase was observed between 2001 and 2004 (APC, +29.4%; p < 0.05), followed by a prolonged period of relative stability from 2004 to 2014, during which rates changed minimally and trended slightly downward (APC, -2.2%; p < 0.05). A significant surge occurred between 2014 and 2018 (APC, +54.2%; p < 0.05), resulting in a nearly 10-fold increase compared to early 2000s levels. Although rates continued to increase from 2018 to 2020 (APC, +8.6%; p > 0.05), this did not reach statistical significance (Figures 12, 13). Benzodiazepine-related mortality increased significantly from 0.3 to 4.8 per 100,000, corresponding to an APC of +14.3% (p < 0.05) (Figures 12, 14).

## Discussion

Our analysis of temporal trends in drug overdose mortality in Pennsylvania from 1999 to 2020 reveals a rapidly evolving epidemic characterized by steep increases across all adult age groups, widening racial and ethnic disparities, and a shift toward a high-risk polysubstance environment. While overdose mortality rose substantially for the entire population, the degree of burden has become increasingly concentrated among young and middle-aged adults, particularly those aged 25-54 years. Furthermore, the demographic profile of the epidemic has fundamentally shifted. What was once characterized largely by mortality among non-Hispanic White populations has transformed into a crisis disproportionately affecting non-Hispanic Black and Hispanic residents, with non-Hispanic Black individuals experiencing a more than fivefold increase in mortality rates. These demographic shifts are underpinned by parallel and accelerating rises in deaths involving opioids, cocaine, and benzodiazepines. This suggests that the current landscape is driven not by a single substance but by complex polysubstance combinations.

Our findings regarding the concentration of overdose mortality among adults aged 25-54 years align with national surveillance data, which consistently identify this demographic as bearing the highest burden of the opioid crisis. A national data brief covering 1999-2020 similarly reported that adults aged 35-44 years had the highest overdose death rates, followed closely by those aged 25-34 years [10]. This concentration of mortality in working-age adults results in a profound loss of potential life years. In 2016 alone, opioid-related deaths accounted for over 1.6 million years of life lost (YLL), with the highest burden per capita falling on adults aged 25-34 years [1]. The accelerated mortality observed in midlife adults also mirrors prior findings that documented a marked increase in drug-poisoning death rates among US adults aged 35-49 between 2013 and 2017 [11].

Recent national surveillance suggests stabilization or modest declines in overall overdose mortality beginning in 2021, although age-specific declines have not been confirmed in the finalized national data [12,13]. A study of Medicaid beneficiaries found that young adults aged 18-34 years who survived a nonfatal opioid overdose had an all-cause mortality rate nearly 40 times higher than the general population [14]. Together, these findings suggest that the mortality trends observed in Pennsylvania likely represent only the tip of the iceberg of a broader crisis affecting the health and longevity of young adults.

Perhaps the most critical finding of our study is the dramatic escalation in overdose mortality among non-Hispanic Black individuals, particularly after 2014. This mirrors a national inversion of historical racial disparities [15]. A study evaluating US overdose mortality before and during the COVID-19 pandemic found that in 2020, the overdose death rate among Black individuals exceeded that of White individuals for the first time since 1999 [16]. Within Pennsylvania specifically, an analysis of hospitalizations during the COVID-19 pandemic corroborated our mortality findings, reporting that while non-Hispanic White patients remained the largest group, the rate of opioid overdose hospitalizations and deaths rose much faster among non-Hispanic Black and Hispanic ethnicity residents [17]. This widening gap is further illustrated by local data from Philadelphia, where fatal overdoses among non-Hispanic Black individuals increased by over 50% following the onset of the pandemic while simultaneously declining among non-Hispanic White individuals [18].

Despite these clear trends, the literature offers some nuance regarding the absolute magnitude of these disparities. A CDC report on adults aged 25-44 years noted that while Black and Hispanic populations experienced the fastest relative increases in overdose deaths between 2019 and 2020, the absolute mortality rate in 2020 remained highest among White adults in that specific age band [19]. This suggests that while the trajectory is most alarming for minority populations, the crisis remains widespread across all racial groups. Research has suggested that neighborhood-level structural racism, including historical redlining and contemporary economic segregation, is strongly associated with higher rates of fatal opioid overdoses [20]. Additionally, disparities in access to life-saving interventions play a crucial role. A study of Medicare beneficiaries found that Black and Hispanic patients were significantly less likely than White patients to receive buprenorphine or naloxone following an opioid-related index event [21]. Similarly, a survey of the naloxone care cascade revealed that Black adults had substantially lower odds of naloxone training and possession compared to White adults [22]. These structural and access barriers are believed to contribute to the disproportionate mortality burden we also observed in Pennsylvania’s Black and Hispanic communities.

The concurrent rise in opioid-, cocaine-, and benzodiazepine-related mortality in our study strongly points to a high-risk polysubstance environment, a trend often described as the “fourth wave” of the overdose epidemic. National analyses have documented that the proportion of overdose deaths involving both fentanyl and stimulants increased dramatically starting around 2015 [23]. A study of US overdose deaths found that by 2019, approximately 76% of cocaine-involved deaths also involved an opioid [24]. This aligns with our finding of a sharp acceleration in cocaine-related mortality in Pennsylvania after 2014. The causes for this finding are theorized to be a mix of intentional co-use and the contamination of the drug supply. A study of one million urine drug test specimens found that the rate of nonprescribed fentanyl positivity among cocaine-positive samples increased by 1850% between 2013 and 2018 [25]. This indicates that many individuals using stimulants may be unknowingly exposed to lethal doses of fentanyl.

Interestingly, our finding of rising benzodiazepine-related mortality in Pennsylvania contrasts with some earlier national data. A study covering 1996-2013 reported that benzodiazepine overdose death rates appeared to plateau after 2010 [26]. The continued increase we observed through 2020 likely reflected the changing composition of the illicit drug supply in the fentanyl era rather than prescribing trends alone. Canadian seizure data from 2018 to 2021 demonstrated a rapid rise in the detection of benzodiazepines within synthetic opioid samples, suggesting that the so-called “benzo-dope” combinations are becoming more prevalent [27]. This is clinically significant because benzodiazepines do not respond to naloxone, potentially complicating overdose reversal efforts. Furthermore, clinical data from emergency departments show that psychostimulant co-ingestion is present in more than half of nonfatal opioid overdoses and is associated with higher naloxone dosing requirements [28]. Consequently, the polysubstance mortality trends in Pennsylvania likely reflect a market where opioids are increasingly ubiquitous, appearing in combination with stimulants and sedatives to create a more lethal drug supply.

The clinical and policy implications of these findings for Pennsylvania are substantial. The concentration of deaths among adults aged 25-54 years and the rising rates among youth necessitate age-tailored prevention and treatment strategies that go beyond the traditional focus on prescription opioids. Moreover, the profound racial disparities highlight an urgent need for equity-focused interventions. Intervention programs and statewide naloxone distribution initiatives must prioritize high-burden Black and Hispanic communities, ensuring that harm reduction tools and medications for opioid use disorder are accessible and culturally responsive. The structural nature of these disparities argues for interventions that also address social determinants of health. Additionally, the clear signal of polysubstance mortality requires that clinical toxicology screening and public health messaging address the risks of fentanyl contamination in non-opioid drugs, including cocaine and counterfeit pills.

Limitations

Our study has several strengths, including the use of population-level data from the entire state of Pennsylvania over a 22-year period, which enables the detection of long-term trends and inflection points. The use of standardized ICD-10 coding for specific substances enables a granular view of the changing overdose landscape. However, limitations should also be acknowledged. First, reliance on death certificate data is subject to misclassification. A study of incomplete death certificates in Pennsylvania suggested that opioid-related deaths may be substantially undercounted due to the use of unspecified drug codes [29]. Second, we could not assess nonfatal overdoses, which are critical for understanding the full burden of morbidity. Third, the ecological nature of the analysis prevents us from making causal inferences about the specific drivers of the observed trends or the direct impact of state policies. In addition, our analysis did not include geographic stratification within Pennsylvania; therefore, potential differences in overdose mortality trends across urban, suburban, and rural communities could not be evaluated. Finally, the study period ended in 2020 and therefore does not capture more recent changes in the illicit drug supply, including the emergence of xylazine-adulterated fentanyl, which has become an increasingly important contributor to overdose morbidity and mortality in Pennsylvania. These factors should be considered when interpreting the findings and may limit generalizability to other populations or to the current overdose landscape.

## Conclusions

In conclusion, drug overdose mortality in Pennsylvania has reached crisis levels characterized by extreme heterogeneity across age and race, driven by a volatile polysubstance market. The epidemic has evolved from one centered on prescription opioids among White populations to one involving fentanyl, stimulants, and benzodiazepines that disproportionately kill young and middle-aged adults and Black and Hispanic residents. These findings underscore that the one-size-fits-all approach to overdose prevention is no longer viable. Future efforts must integrate the real-time surveillance of drug supply composition with targeted, community-led interventions that address the specific structural and clinical barriers faced by the populations most at risk.

## References

[1] Gomes T, Tadrous M, Mamdani MM, Paterson JM, Juurlink DN (2018). The burden of opioid-related mortality in the United States. JAMA Netw Open.

[2] Spencer MR, Miniño AM, Warner M (2022). Drug overdose deaths in the United States, 2001-2021. 2001-2021. NCHS Data Brief.

[3] Salazar CI, Huang Y (2022). The burden of opioid-related mortality in Texas, 1999 to 2019. Ann Epidemiol.

[4] Althoff KN, Leifheit KM, Park JN, Chandran A, Sherman SG (2020). Opioid-related overdose mortality in the era of fentanyl: monitoring a shifting epidemic by person, place, and time. Drug Alcohol Depend.

[5] Kariisa M, Scholl L, Wilson N, Seth P, Hoots B (2019). Drug overdose deaths involving cocaine and psychostimulants with abuse potential - United States, 2003-2017. MMWR Morb Mortal Wkly Rep.

[6] Kariisa M, Seth P, Scholl L, Wilson N, Davis NL (2021). Drug overdose deaths involving cocaine and psychostimulants with abuse potential among racial and ethnic groups - United States, 2004-2019. Drug Alcohol Depend.

[7] Brinzo PN, Martins SS (2024). Racial/ethnic trends in opioid and polysubstance opioid overdose mortality in adolescents and young adults, 1999-2020. Addict Behav.

[8] Holmes LM, Rishworth A, King BH (2022). Disparities in opioid overdose survival and naloxone administration in Pennsylvania. Drug Alcohol Depend.

[9] (2026). National Center for Health Statistics: mortality data on CDC WONDER. https://wonder.cdc.gov/mcd.html.

[10] (2021). Drug overdose deaths in the United States, 1999-2020. https://stacks.cdc.gov/view/cdc/112340.

[11] Shiels MS, Tatalovich Z, Chen Y (2020). Trends in mortality from drug poisonings, suicide, and alcohol-induced deaths in the United States from 2000 to 2017. JAMA Netw Open.

[12] Kariisa M, O'Donnell J, Kumar S, Mattson CL, Goldberger BA (2023). Illicitly manufactured fentanyl-involved overdose deaths with detected xylazine — United States, January 2019-June 2022. Morb Mortal Wkly Rep.

[13] Tanz LJ, Stewart A, Gladden RM, Ko JY, Owens L, O'Donnell J (2024). Detection of illegally manufactured fentanyls and carfentanil in drug overdose deaths — United States, 2021-2024. Morb Mortal Wkly Rep.

[14] Olfson M, Crystal S, Wall M, Wang S, Liu SM, Blanco C (2018). Causes of death after nonfatal opioid overdose. JAMA Psychiatry.

[15] Lippold K, Ali B (2020). Racial/ethnic differences in opioid-involved overdose deaths across metropolitan and non-metropolitan areas in the United States, 1999-2017. Drug Alcohol Depend.

[16] Friedman J, Shover CL (2023). Charting the fourth wave: geographic, temporal, race/ethnicity and demographic trends in polysubstance fentanyl overdose deaths in the United States, 2010-2021. Addiction.

[17] Shen C, Thornton JD, Li N, Zhou S, Wang L, Leslie DL, Kawasaki SS (2024). Opioid overdose hospitalizations during COVID-19: the experience of Pennsylvania. Subst Use.

[18] Khatri UG, Pizzicato LN, Viner K, Bobyock E, Sun M, Meisel ZF, South EC (2021). Racial/ethnic disparities in unintentional fatal and nonfatal emergency medical services-attended opioid overdoses during the COVID-19 pandemic in Philadelphia. JAMA Netw Open.

[19] (2022). Quickstats: death rates for drug overdose* among persons aged 25-44 years, by race and ethnicity†— United States, 2000-2020. MMWR Morbidity and Mortality Weekly Report [Internet.

[20] Barnett ML, Meara E, Lewinson T (2023). Racial inequality in receipt of medications for opioid use disorder. N Engl J Med.

[21] Uzzi M, Ricard JR, Belton I (2025). Fatal opioid overdoses by historical and contemporary neighborhood-level structural racism. JAMA Health Forum.

[22] Allen L, Black JC, Kelly CM (2025). Black, Hispanic, and Asian adults in the US had substantially lower engagement on the naloxone care cascade, 2024. Health Aff (Millwood).

[23] Friedman JR, Hansen H (2022). Evaluation of increases in drug overdose mortality rates in the US by race and ethnicity before and during the COVID-19 pandemic. JAMA Psychiatry.

[24] Hedegaard H, Miniño AM, Warner M (2020). Drug overdose deaths in the United States, 1999-2019. NCHS Data Brief.

[25] LaRue L, Twillman RK, Dawson E, Whitley P, Frasco MA, Huskey A, Guevara MG (2019). Rate of fentanyl positivity among urine drug test results positive for cocaine or methamphetamine. JAMA Netw Open.

[26] Bachhuber MA, Hennessy S, Cunningham CO, Starrels JL (2016). Increasing benzodiazepine prescriptions and overdose mortality in the United States, 1996-2013. Am J Public Health.

[27] Pardo B (2022). Insights into mixing fentanyl and benzodiazepines from Canadian drug seizures. JAMA Psychiatry.

[28] Shastry S, Shulman J, Aldy K, Brent J, Wax P, Manini AF (2024). Psychostimulant drug co-ingestion in non-fatal opioid overdose. Drug Alcohol Depend Rep.

[29] Buchanich JM, Balmert LC, Williams KE, Burke DS (2018). The effect of incomplete death certificates on estimates of unintentional opioid-related overdose deaths in the United States, 1999-2015. Public Health Rep.

